# Retention and Attrition in the Psychiatry Workforce: A Scoping Review

**DOI:** 10.1192/bjo.2026.11302

**Published:** 2026-06-30

**Authors:** Jun Jie Lim, Cherise Jensen, Oscar Taylor, Adrian Lloyd, Hugh Alberti

**Affiliations:** 1 School of Medicine, Newcastle University, Newcastle upon Tyne, United Kingdom; 2 Cumbria, Northumberland, Tyne and Wear NHS Foundation Trust, Newcastle upon Tyne, United Kingdom; 3 Independent Researcher, formerly School of Medicine, Newcastle University Medical School, Newcastle upon Tyne, United Kingdom; 4 School of Medicine, University of Sunderland, Sunderland, United Kingdom; 5 Health Education England, Newcastle upon Tyne, United Kingdom

## Abstract

**Aims:**

The psychiatry workforce faces persistent shortages driven by high attrition and poor retention across training and consultant grades. This scoping review aimed to map the existing evidence on psychiatry workforce retention and attrition, identify factors influencing decisions to stay or leave, and examine interventions, strategies, or policies designed to address workforce instability.

**Methods:**

We conducted a scoping review following Arksey and O’Malley’s five-stage framework, reported in line with PRISMA-ScR guidance. MEDLINE, EMBASE, PsycINFO, Scopus, and Web of Science were searched alongside grey literature sources. Studies addressing retention, attrition, turnover, progression, or intention to stay or leave among psychiatrists or psychiatry trainees were included. Data were synthesised using descriptive mapping and inductive thematic analysis.

**Results:**

Screening identified 4110 articles. Of 193 articles selected for full-text review, 65 articles were included. Attrition was consistently associated with excessive workload,burnout, inadequate staffing, unsafe working environments, administrative burden, and poor supervision or training quality. Retention was linked to supportive supervision, meaningful clinical work, flexible job design, professional development, and a sense of belonging. Migration decisions were shaped by systemic push factors, including low pay and under-resourced services, and pull factors such as better working conditions, training quality, and social stability. Few proposed retention strategies had been formally evaluated.

**Conclusion:**

The psychiatry workforce is characterised by substantial instability driven by multi-level factors spanning individual, organisational, and system domains. Although many retention strategies are proposed, robust evaluation is lacking. Addressing workforce sustainability will require system-level investment and evidence-based interventions targeting workload, training environments, supervision, and career flexibility.

